# The Electrochemical Oxidation and Mass Transfer Mechanism of Formic Acid on the Catalyst Electrode Surface

**DOI:** 10.3389/fchem.2022.914699

**Published:** 2022-06-13

**Authors:** Jie Hu, Panpan Wang, Changguo Chen

**Affiliations:** ^1^ Department of Environment and Quality Test, Chongqing Chemical Industry Vocational College, Chongqing, China; ^2^ China Merchants Testing Vehicle Technology Research Institute Co., Ltd., Chongqing, China; ^3^ College of Chemistry and Chemical Engineering, Chongqing University, Chongqing, China

**Keywords:** adhesion, crystal structure, interfaces, catalyst, nucleation process

## Abstract

The organic small molecule fuel battery has attracted wild attention in recent years. Unfortunately, the inherent catalyst poisoning phenomenon hinders its commercialization. Exploring the anodic catalytic reaction mechanism is urgent. This article investigates the nucleation mechanism of HCOOH on the catalyst electrode surface. The electrochemical results indicate that the HCOOH oxidation conforms to the two-dimensional instantaneous nucleation process. The corresponding adsorption model of CO on the catalyst surface was finally established.

## Introduction

Developing clean energy is the key to solving environmental pollution and energy crisis problems (Xu et al., 2020a; Shuai et al., 2020; Xu et al., 2021). The organic small molecule fuel battery has attracted wild attention in recent years due to its high efficiency, friendly environment and so on (Xu et al., 2020b). However, the electrochemical oxidation process and the catalyst poisoning phenomenon of organic molecules are unclear, severely restricting its development. Thus, it is necessary to develop research on the anodic reaction mechanism of organic small molecules on the catalyst electrode surface, which can provide a new idea to improve the fuel battery efficiency.

The electrocatalytic oxidation reaction of formic acid (HCOOH) on the metal surface is often used as a reaction model to study the structure-performance relationship of other complex catalytic systems due to its simple molecular structure and favorable sensitivity on the electrocatalyst surface (Chen et al., 2006a). In recent years, various electrochemical techniques have been used to study the effect of different factors on the electrocatalytic oxidation mechanism of HCOOH (Samjeské et al., 2005; Chen et al., 2006b; Mukouyama et al., 2006; Osawa et al., 2011). In addition, the calculation of HCOOH oxidation based on DFT theory has also been developed rapidly (Wang and Liu, 2015). Parson proposed that the oxidation of formic acid on the catalyst electrode surface may follow a dual pathway mechanism, namely, the indirect pathway and the direct pathway (Capon and Parsons, 1973). In the direct pathway, formic acid removes H atomic to produce CO_2_. In the indirect pathway, formic acid first dehydrates to produce CO toxic intermediate, closely adsorbed on the electrode surface, and CO is oxidized to CO_2_ with the increase in the electric potential (Watanabe and Motoo, 1975).

Although there are many studies on the electrochemical oxidation mechanism of HCOOH on the catalyst electrode surface, they mainly focus on detecting the dissociation of adsorbed species of organic small molecules on the electrode surface. The reports of nucleation/growth dynamics are very few, which can provide abundant information about the molecular mechanism with the electrode surface. The phenomenon of the solid-phase transition is widespread in the electrode surface, such as metal electrodeposition, Ag_2_O and AgO transformation, and adsorption-desorption process (Gómez et al., 1992). Sun et al. obtained the *i*-*t* transient current curves of HCOOH oxidation on catalyst electrode surface at different adsorption times using the electrical step experiment. However, they did not discuss the nucleation and growth kinetics of adsorptive species on the catalyst electrode surface (Sun et al., 1992). Therefore, it is necessary and significant to explore the nucleation mechanism and kinetic process of HCOOH on the catalyst electrode surface. The dimensionless processed transient curves confirmed that the nucleation/growth process of HCOOH on the catalyst electrode surface followed the diffusion-controlled two-dimensional (2D) instantaneous nucleation model. This groundbreaking research can help us understand the redox process mechanism of HCOOH and provide a theoretical basis for choosing high-performance fuel battery catalysts.

## Experiment

**SCHEME 1:**
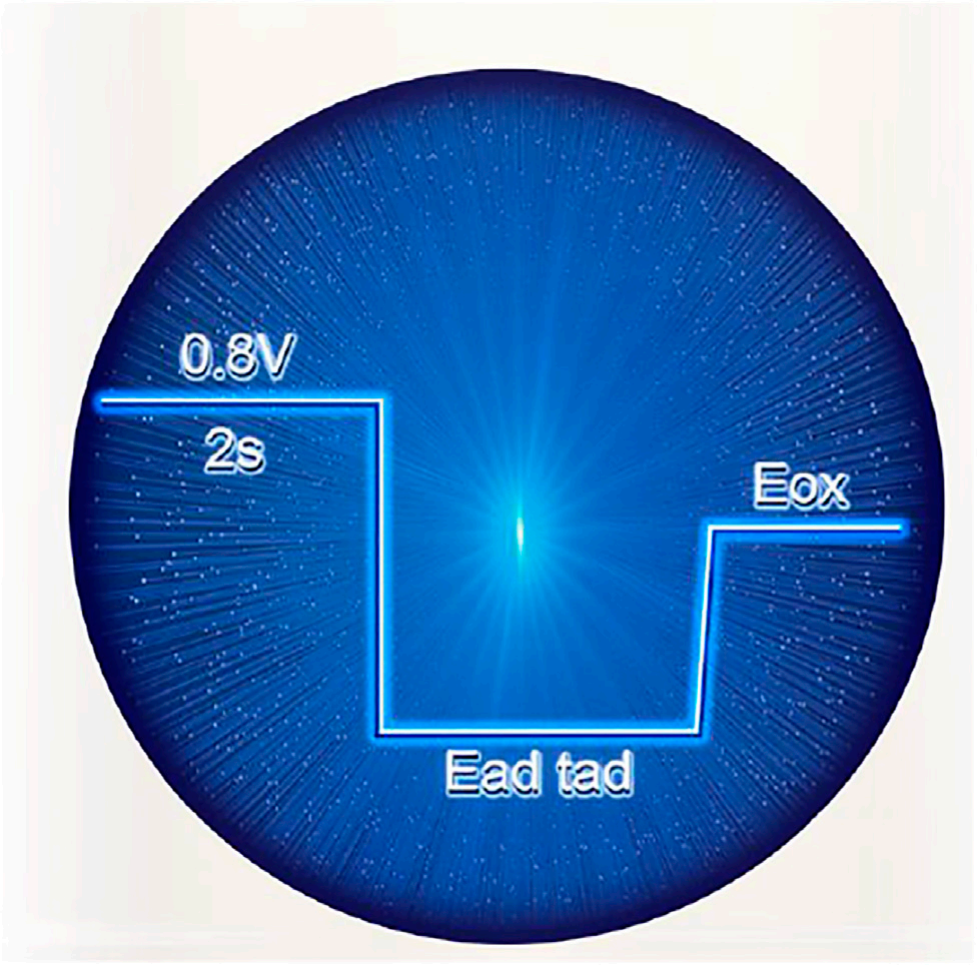
Multi-potential step parameter.

All chemicals were of analytical grade. The testing solution is 0.5 mmol/L HCOOH + 0.1 mmol/L HClO_4_. The electrochemical measurements were conducted using CHI 660E electrochemical workstation at room temperature. A conventional three-electrode system was conducted with catalyst as the counter electrode, Ag/AgCl as the reference electrode, and catalyst electrode as the working electrode. The electric step parameters are as shown in Scheme 1.

## Results and Discussion

### Adsorption Curve Analysis

**FIGURE 1:**
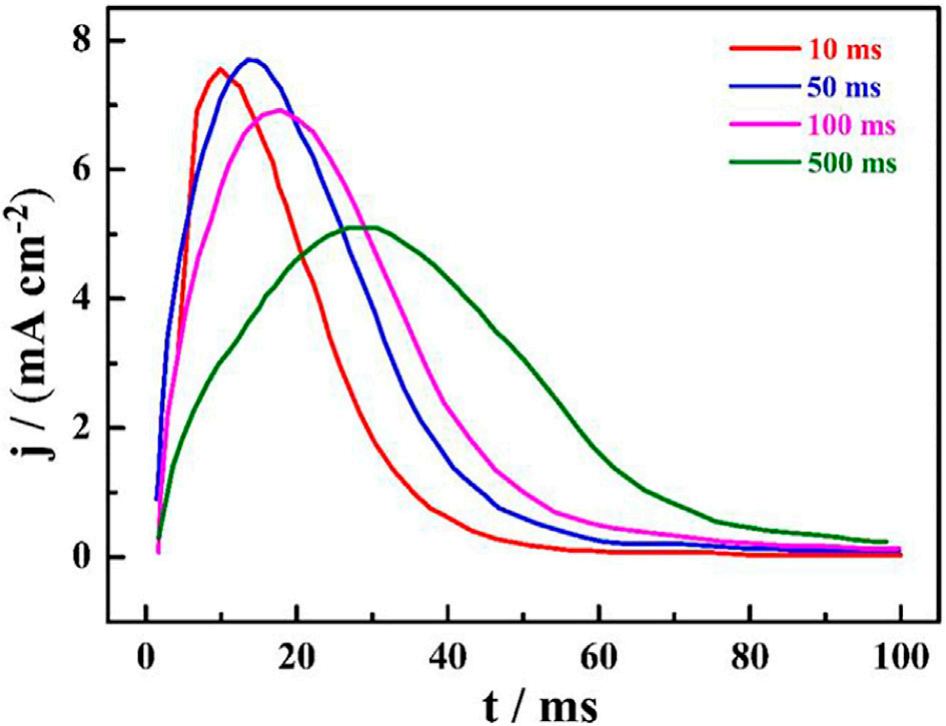
The *i*-*t* net current curves of HCOOH on a single crystal catalyst electrode surface at different adsorption times.

Figure 1 shows the *i*-*t* curves of HCOOH on the catalyze electrode surface, which can reflect the dissociation and adsorption process of the intermediate HCOOH reaction. The net current curves were obtained by subtracting the background current; namely, the adsorption time was 0 ms, which can reflect the oxidation current of the adsorbed CO. It can be found that the oxidation peak became more obvious and the peak type changed from high and thin to low and fat with the increase in the adsorption time. In addition, all oxidation processes were completed within 100 ms, which was related to the low HCOOH concentration.

### Determination of Nucleation Model

**FIGURE 2:**
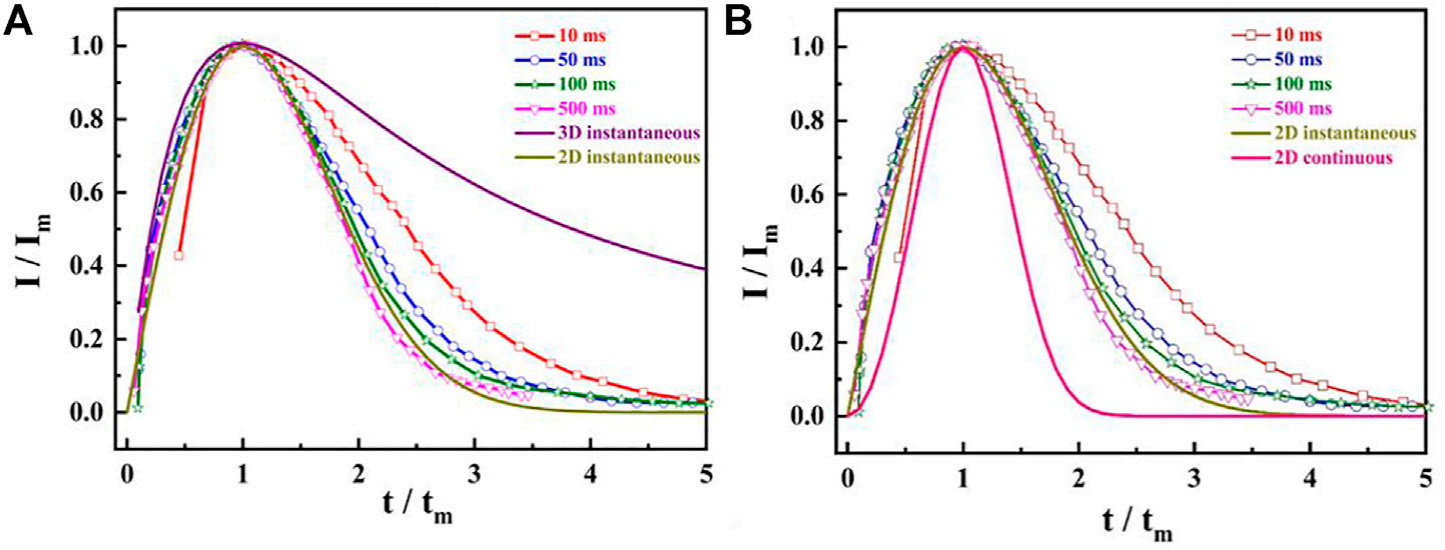
The dimensionless (*I*/*I*_*m*_ ∼ *t*/*t*_*m*_) curves at different adsorption times: **(A)** 2D and 3D instantaneous model; **(B)** 2D instantaneous and continuous model.

Figure 2 shows that the *i*-*t* curves display a typical electrocrystallization nucleation characteristic, which is the appearance of the maximum peak current in the transient curves. Although the mathematical expression models of the metal nucleation are abundant, the most widely used is the dimensionless expression with undetermined parameters in the research process of electrocrystallization mechanism using i-t transient curves, which can be expressed as follows (Fleiu et al., 1994):

J2Jm2=1.9542ttm{1−exp[−1.2546(ttm)]}2 (1)

  J2 Jm2=1.2254ttm{1−exp[−2.3367(t2tm)]}2 (2)

where *I*_*m*_ and *t*_*m*_ denote the maximum current density (A^**.**^cm^−2^) and its corresponding time, respectively. Comparing the obtained *I/I*_*m*_-*t/t*_*m*_ curves with the theoretical nucleation curves obtained in Eqs 1, 2 and confirming the solid phase nucleation mode of CO on the catalyst electrode surface, the experimental results show that the nucleation process of the oxidation intermediates of HCOOH (CO) on the single crystal catalyst electrode surface conforms to the 2D instantaneous nucleation model at any testing time. It is worth noting that the coincidence degree of the obtained curves with the 2D instantaneous nucleation curve is higher with the extension of testing time.

### Dimensionless Curve Fitting

The dimensionless expressions of 2D instantaneous nucleation can be further arranged as follows (Bewick et al., 1962):

$$\ln\left(\frac{I}{t}\right)=A_{1}-B_{1}t^{2}$$ (3)

**FIGURE 3:**
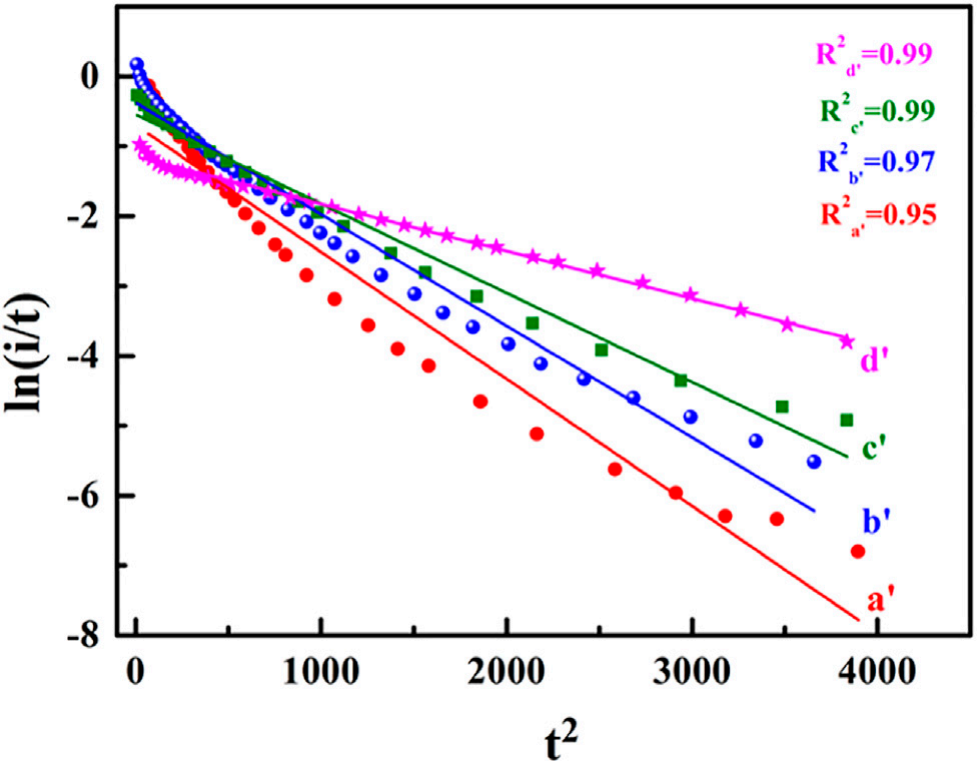
The In (I/t) ∼t^2^ curves of HCOOH on a single crystal catalyst electrode.

To further verify the above experimental conclusion, the experimental data were further processed according to Formula 3, and the results are shown in Figure 3. It can be seen that the In (I/t) ∼t^2^ curves under different adsorption times displayed a good linear relationship with the obtained theoretical curves.

The results further confirmed that the CO oxidation process of HCOOH hydrolysis and adsorption on the surface of the single crystal catalyst electrode was a 2D instantaneous nucleation process, and the curve fitting degree increased with the increase of adsorption time.

### CO Adsorption Model

**FIGURE 4:**
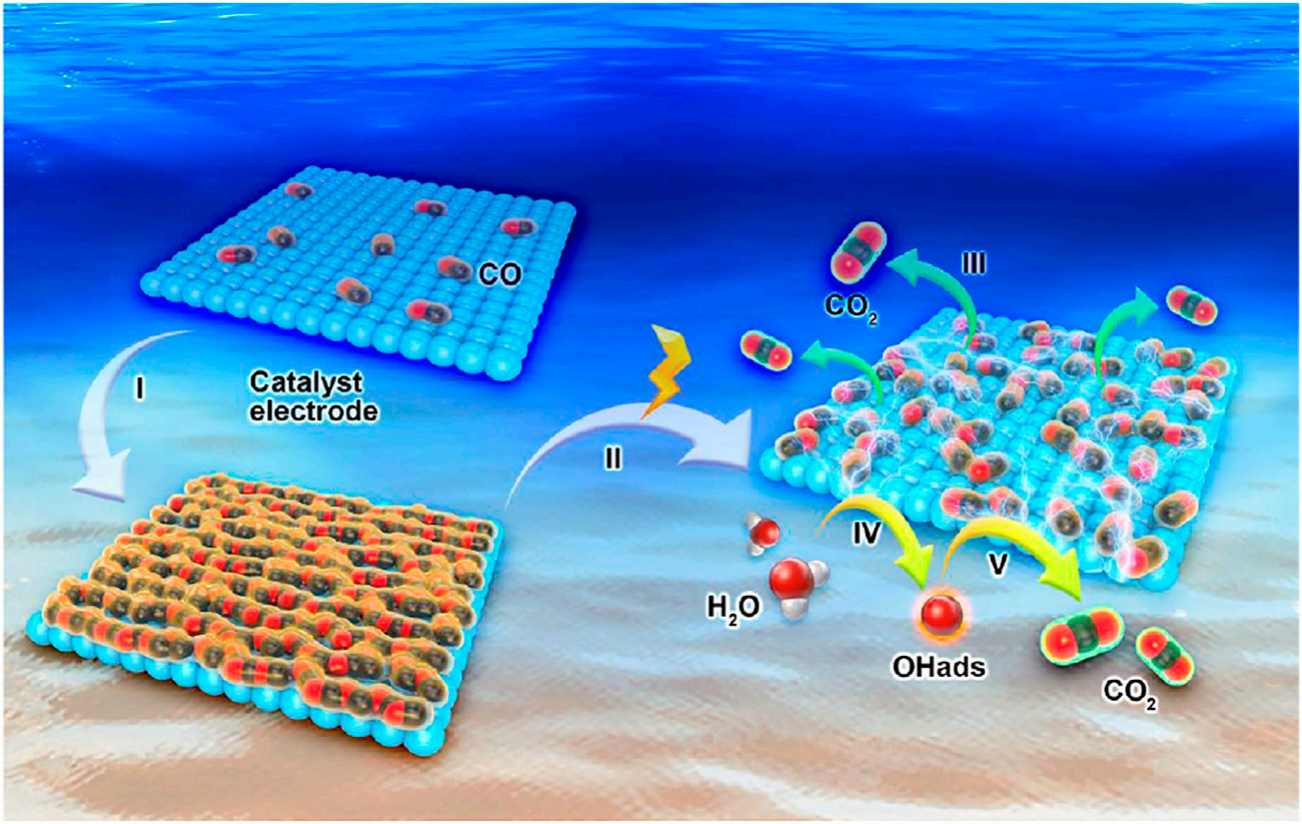
Scheme of the CO adsorption model.

According to the above experimental results, the adsorption model of the HCOOH oxidation intermediate CO at the catalyst electrode surface is shown in Figure 4. Based on the definition of 2D instantaneous nucleation, assuming that the adsorbed CO on the catalyst electrode surface can be seen as the independent island, the small islands could collide fusion and gradually occupy the electrode surface with the extension of adsorption time. Finally, the catalyst electrode surface was completely dominated by CO molecules and lost the catalytic activity, namely, the catalyst poisoning. CO was oxidized to CO_2_ when the applied signal reached oxidation potential, and the catalyst electrode was exposed again to restore the catalytic activity. Moreover, atoms can promote the activation of H_2_O molecules to form OH_ads_ and reaction with CO to form CO_2_ and further promote the HCOOH oxidation on the electrode surface.

## Conclusion

In conclusion, the experimental result indicated that the adsorption of HCOOH oxidation intermediates (CO) on the catalyst electrode surface conformed to the 2D instantaneous nucleation mechanism at any testing adsorption time and the corresponding nucleation model was established. This research can help us understand the redox process mechanism of HCOOH and provide a theoretical basis for choose of high-performance fuel battery catalysts.

## Data Availability

The original contributions presented in the study are included in the article/supplementary material. Further inquiries can be directed to the corresponding authors.
